# Intracranial hemodynamic relationships in patients with cerebral small vessel disease

**DOI:** 10.1212/WNL.0000000000009483

**Published:** 2020-05-04

**Authors:** Gordon W. Blair, Michael J. Thrippleton, Yulu Shi, Iona Hamilton, Michael Stringer, Francesca Chappell, David Alexander Dickie, Peter Andrews, Ian Marshall, Fergus N. Doubal, Joanna M. Wardlaw

**Affiliations:** From the Brain Research Imaging Centre (G.W.B., M.J.T., Y.S., I.H., M.S., F.C., P.A., I.M., F.N.D., J.M.W.), Centre for Clinical Brain Sciences, University of Edinburgh, United Kingdom; UK Dementia Research Institute at The University of Edinburgh (G.W.B., M.J.T., Y.S., I.H., M.S., F.N.D., J.M.W.), Edinburgh Medical School, United Kingdom; Beijing Tiantan Hospital Affiliated to Capital Medical University (Y.S.), China; Institute of Cardiovascular and Medical Sciences (D.A.D.), University of Glasgow, United Kingdom; and Centre for Cognitive Ageing and Cognitive Epidemiology (J.M.W.), University of Edinburgh, United Kingdom.

## Abstract

**Objective:**

To investigate cerebrovascular reactivity (CVR), blood flow, vascular and CSF pulsatility, and their independent relationship with cerebral small vessel disease (SVD) features in patients with minor ischemic stroke and MRI evidence of SVD.

**Methods:**

We recruited patients with minor ischemic stroke and assessed CVR using blood oxygen level–dependent MRI during a hypercapnic challenge, cerebral blood flow (CBF), vascular and CSF pulsatility using phase-contrast MRI, and structural magnetic resonance brain imaging to quantify white matter hyperintensities (WMHs) and perivascular spaces (PVSs). We used multiple regression to identify parameters associated with SVD features, controlling for patient characteristics.

**Results:**

Fifty-three of 60 patients completed the study with a full data set (age 68.0% ± 8.8 years, 74% male, 75% hypertensive). After controlling for age, sex, and systolic blood pressure, lower white matter CVR was associated with higher WMH volume (−0.01%/mm Hg per log10 increase in WMH volume, *p* = 0.02), basal ganglia PVS (−0.01%/mm Hg per point increase in the PVS score, *p* = 0.02), and higher venous pulsatility (superior sagittal sinus −0.03%/mm Hg, *p* = 0.02, per unit increase in the pulsatility index) but not with CBF (*p* = 0.58). Lower foramen magnum CSF stroke volume was associated with worse white matter CVR (0.04%/mm Hg per mL increase in stroke volume, *p* = 0.04) and more severe basal ganglia PVS (*p* = 0.09).

**Conclusions:**

Lower CVR, higher venous pulsatility, and lower foramen magnum CSF stroke volume indicate that dynamic vascular dysfunctions underpin PVS dysfunction and WMH development. Further exploration of microvascular dysfunction and CSF dynamics may uncover new mechanisms and intervention targets to reduce SVD lesion development, cognitive decline, and stroke.

Cerebral small vessel disease (SVD) causes multiple major health problems including lacunar ischemic stroke, vascular dementia, and intracerebral hemorrhage.^1^ There is no specific treatment for SVD.^2^ An improved understanding of disease mechanisms is required to identify drug targets.

The role of cerebral blood flow (CBF) in SVD is complex: although lower CBF is associated with more severe SVD in cross-sectional studies, most longitudinal studies have not found lower CBF to precede development or progression of SVD.^3^ Dynamic measures of CBF may be more sensitive to small vessel dysfunction.

Cerebrovascular reactivity (CVR) measures the vasculature's ability to increase CBF in response to metabolic demands.^4^ CVR can be assessed by measuring blood flow changes in response to a vasoactive stimulus such as breathing carbon dioxide.^5^ Transcranial Doppler (TCD) ultrasound is commonly used but only measures large cerebral arteries.^6,7^ MRI techniques measure CVR in all brain tissues, including the subcortical areas commonly affected by SVD.^8^ So far, MRI-based CVR has been assessed in relatively few small studies in SVD, but used different CVR challenges,^4,9–12^ and did not account for potentially confounding clinically relevant variables (e.g., age, blood pressure [BP], and sex).^4^

More pulsatile blood flow (larger difference in blood flow between systolic and diastolic phases) measured outside the brain is associated with increasing white matter hyperintensities (WMHs)^13,14^ consistent with the hypothesis that SVD is associated with increased arterial stiffening. MRI phase-contrast angiography can assess pulsatility and flow in the intracranial arteries, venous sinuses, and CSF spaces (aqueduct and foramen magnum) within 1 examination, concurrently with CVR. However, there are very few studies of intracranial pulsatility assessed with MRI in aging or SVD^15^ and no studies in humans of CVR assessed concurrently with intracranial vascular pulsatility, CSF flow dynamics, or resting CBF.

We aimed to assess CVR and imaging features of SVD, concurrently with total CBF, arterial, venous, and CSF pulsatility. We studied independent patients with minor ischemic stroke stratified by SVD burden as representative of a high-risk group for the clinical effects of SVD. We hypothesized that more severe SVD features on neuroimaging would be associated with lower CVR, that lower CVR would associate with increased vascular pulsatility, and that these relationships would persist after controlling for patient demographic and vascular risk factors.

## Methods

### Standard protocol approvals, registrations, and patient consents

All participants provided written informed consent before enrollment. The study was approved by the UK Health Research Authority National Research Ethics Service Committee East Midlands, Nottingham 1 (ref. 14/EM/1126).

### Patients

We invited all patients presenting to our regional stroke service between October 2014 and April 2016 with symptomatic nondisabling ischemic stroke (modified Rankin Scale score ≤3^16^) and whose treating physician agreed could be approached about the study to take part in the study. We also invited such patients who had participated in recent previous prospective studies.^17^ All patients had a clinical stroke diagnosis confirmed by a specialist stroke physician and brain imaging that either confirmed a relevant recent infarct or, if no recent infarct was visible, excluded any other cause of the presenting symptoms.^17^

We excluded patients who were pregnant, unable to lie flat, had contraindications to MRI (including claustrophobia), moderate to severe chronic respiratory disease or symptomatic cardiac failure, personal history or first-degree relative with subarachnoid hemorrhage or intracranial aneurysm, uncontrolled hypertension, or atrial fibrillation with fast ventricular response.

We performed the study assessments at least 1 month after the patient's stroke to prevent hemodynamic changes in the acute phase of stroke (both stroke related and due to commencing vasoactive secondary prevention medications) from interfering with the interpretation of vascular function. We requested participants not to consume caffeinated drinks on the day of assessment.

We recorded detailed medical histories including clinical characteristics of presenting stroke, vascular risk factors, smoking and alcohol use (with alcohol excess defined as consuming more than 21 units per week), concurrent medications, and investigations performed for the presenting stroke (diagnostic brain MRI, carotid ultrasound imaging, hematology, biochemistry, and ECGs).

We classified the clinical stroke syndrome according to the Oxford Community Stroke Project classification^18^ (independently assessed by 2 stroke physicians, F.N.D. and G.W.B.) and the lesion type seen on imaging (independently assessed by J.M.W. and Y.S.). Any disagreements were resolved by discussion. Where no recent ischemic lesion was evident, the final stroke type was assigned using the clinical classification. Where the imaging lesion was discordant with the clinical stroke type, the imaging type was used as the final classification, as previously.^19^ We recorded BP 7 times at specific points of the study visit that included measurements before, during, and after MRI.

### MRI

We performed brain MRI using a 1.5 Tesla GE research MRI scanner (Signa HDxt; General Electric, Milwaukee, WI) operating in research mode and an 8-channel phased array head coil. The total imaging time was circa 75 minutes for each participant, but patients were allowed to move and have a natural break between each section of scans to ensure that they remained comfortable. We acquired 3D T1-weighted, axial T2-weighted, fluid-attenuated inversion recovery (FLAIR), and gradient echo sequences. We performed blood oxygen level–dependent (BOLD) MRI scanning with CO_2_ challenge at 4 mm isotropic resolution acquiring a whole-brain volume every 3 seconds.^8^ We performed phase-contrast MRI to measure pulsatility in the internal carotid arteries, intracranial venous sinuses, CSF flow (aqueduct and foramen magnum), and total CBF, as described in detail.^23^ Full parameters of all MR sequences are available from Dryad (table e-1, doi.org/10.5061/dryad.xpnvx0kb3).

The CVR method used BOLD 2-dimensional echo-planar imaging with a CO_2_ challenge (detailed in Ref. 8). Briefly, during MRI, patients wore an anesthetic face mask, carefully fitted to avoid gas leak between mask and face, attached to a bespoke unidirectional breathing circuit (Intersurgical, Wokingham, United Kingdom). Monitoring equipment recorded the pulse rate, oxygen saturation, BP (Millennia 3155A and Magnitude 3150 MRI; Invivo, Best, The Netherlands), and end-tidal CO_2_ (EtCO_2_; CD3-A AEI Technologies, Pittsburgh, PA) throughout the examination. During a 12-minute BOLD MRI scan, patients breathed medical air and 6% CO_2_ in air (BOC Special Products; Guildford, United Kingdom) alternately, delivered as 2 minutes air, 3 minutes CO_2_, 2 minutes air, 3 minutes CO_2_, and finishing with 2 minutes air. We instructed patients to expect a change in smell and breathing pattern (deeper, faster, or more forceful breathing), and then each patient tried the facemask and gases before entering the scanner room. Patients were instructed to breathe normally and to press a buzzer to stop the scan if required. Scanning commenced when the patient was fully positioned in the scanner bore, indicated that they felt comfortable, and BP and heart rate readings indicated no sign of anxiety or distress. A physician monitored the patient during CO_2_ inhalation.

Arterial and venous flow waveforms were measured as described previously.^20–22^ Briefly, we used a 2D cine phase-contrast sequence with retrospective peripheral pulse gating to acquire 32 velocity images per cardiac cycle. We used the following slice locations to measure flow in the different structures: a slice superior to the carotid bifurcation and perpendicular to the internal carotid artery (ICA) walls to measure flow in the ICA, vertebral arteries (VAs), and internal jugular veins (IJVs); a coronal-oblique slice intersecting the superior sagittal sinus (SSS) approximately 2 cm above the torcular and through the midpoint of the straight sinus (StS) to measure SSS, StS, and transverse sinus (TS) flow; a slice perpendicular to the aqueduct for aqueduct CSF flow; and an axial slice at the craniocervical junction for foramen magnum CSF flow.

### Image processing and analysis

For each analysis, investigators were blinded to participant demographic and clinical data and other outcome data (e.g., investigators rating imaging features were blinded to CVR data). Structural image analysis of SVD features was performed according to the STRIVE criteria^23^ under the supervision of an expert neuroradiologist (J.M.W.).

We (Y.S. and J.M.W.) scored the following features: WMHs using the Fazekas scale,^24^ summing periventricular and deep WMH scores to give a score from 0 to 6; perivascular spaces (PVSs), scored separately in the basal ganglia and centrum semiovale, using a validated, semiquantitative ordinal scale (range 0–4); lacunes (location and number); and microbleeds (BOMBS scale) presence/absence and total number^23^; atrophy score by reference to a normative age template^25^; and total SVD score (0–4) by combining WMHs, lacunes, microbleeds, and PVS scores, as described previously.^26^

We coregistered each participant's structural images. We calculated WMH volumes using a validated semiquantitative technique described previously.^22^ Briefly, we generated WMH probability maps for each participant using FLAIR and T1W image data. Hyperintense outliers within the white matter surface were defined as voxels with a z score of ≥1.5 on FLAIR to create an initial estimate of WMH volume. We produced final estimates using 3D smoothing to account for partial volume effects and reduce noise before manually removing the index and any previous stroke lesions. We segmented normal-appearing tissues (cortical gray matter, subcortical gray matter, white matter, and cerebellum) and whole-brain volume from each participant's T1W data and local population-specific probability maps.^27^ We calculated intracranial volumes using a semiautomatic method based on T2*W images. All tissue masks were visually inspected and manually corrected as necessary.

We processed CVR images as described previously.^8^ We generated voxel-wise CVR maps by regressing the BOLD signal against EtCO_2_ and the BOLD scan number (to account for signal drift). CVR is expressed as % BOLD signal change/mm Hg change in EtCO_2_, based on the EtCO_2_ regressor in the model. We made additional adjustment for the delay time between BOLD signal change and EtCO_2_ change to minimize the residual sum of squares individually for each voxel, further adjusted by 4 seconds to account for the delay between exhalation and detection on the EtCO_2_ monitor caused by the 8-m-long EtCO_2_ sample tubing.

We realigned BOLD images (using SPM 8, fil.ion.ucl.ac.uk/spm/software/spm8/) before determining the transformation between BOLD and T2W image spaces (using FSL FLIRT^28^). We then manually drew 3 subcortical gray matter (thalamus, putamen, and caudate head) and 4 subcortical white matter (frontal, posterior, periventricular, and centrum semiovale) regions of interest (ROIs) on T1W images before transfer to the BOLD images. Voxels that were part of large vessels or the patient's stroke lesion were manually excluded. We extracted the mean signal across the ROIs and fitted the CVR model to that data. We also averaged all gray and white matter regions to give a combined deep gray matter and white matter CVR value.

To process the phase-contrast data, we drew manual ROIs around the right and left ICAs and VAs; the sagittal, straight, right, and left TSs and IJVs; the aqueduct; and the foramen magnum subarachnoid space. Background ROIs were placed close to the ROIs to correct background phase error by subtracting the background velocity (noise) from the ROI velocity. We calculated sum flow and mean velocity for bilateral structures, then total CBF as the sum of ICA and VA flow, normalized to total brain volume and expressed as mL/min/100 mL brain tissue. Pulsatility index (PI) in each structure was calculated as (Flow_maximum_ − Flow_minimum_)/Flow_mean_; resistivity index (RI) was calculated as (Flow_maximum_ − Flow_minimum_)/Flow_maximum_, with higher values indicating more pulsatile or more resistive blood flow. The reproducibility of this approach is published.^22^ CSF flow defined the net flow in the aqueduct and foramen magnum, respectively. We calculated this by integrating the cranial (positive) and caudal (negative) flow values and expressed the net flow in mL/min. CSF stroke volume reflects the total volume of CSF flow per cardiac cycle and is calculated by averaging the absolute cranial and caudal flow across the cardiac cycles. These methods for CSF data processing were similar to those reported in a previous study.^29^

### Sample size and statistical analysis

Only limited data existed to calculate sample size, however we estimated detecting a relative difference in CVR of 25% between those with low vs high WMH burden based on peripheral vascular function data. Allowing for a 10% scan failure rate, we aimed to recruit 60 participants.

We performed statistical analyses once the study data set was finalized and the database locked for editing, using R version 3.3.0 (cran.r-project.org/) and the additional packages Hmisc, texreg, data.table, htmlTable, car, and psych. We assessed distribution of all variables before analysis and log transformed the WMH volumes and atrophy scores due to a skewed distribution. The BP values presented are means of the 7 readings taken across the visit. We assessed the relation of demographic factors, SVD features, pulsatility, and CBF parameters to CVR values using univariate (for comparison with prior studies) and then multiple linear regression, adjusting for age, systolic BP, and sex, based on those factors identified in our systematic review as potentially influencing CVR^4^ (univariate associations are presented only for completeness). In addition, we adjusted for WMH volume in some models to assess whether relationships were independent of a coassociation because we found previously that failure to control for WMH was a potential confound in assessments of CVR in patients with SVD.^30^ We examined the normality of residuals (QQ plots and histograms) and heteroscedasticity (residual vs fitted values) to assess modeling assumptions. We checked variable inflation factors to assess for collinearity between variables, with a limit of 2 applied.^31^

### Data availability

The full individual patient data anonymized data set, along with the study protocol, is available to bona fide researchers via the University of Edinburgh's Cerebrovascular Diseases Database. Access request should be submitted to J.M. Wardlaw along with a description of any planned analyses.

## Results

### Study population

We recruited 60 patients; 53 participants completed the full CVR scanning protocol with complete and fully analyzable data. Three withdrew due to claustrophobia when wearing the facemask in the MRI scanner, in 1 movement artifact precluded analysis, 2 failed to show any EtCO_2_ or BOLD signal change with CO_2_ (likely due to a poor fitting mask), and in 1 patient, the acquisition was stopped after initial structural scanning showed an incidental asymptomatic subdural hematoma.

The 53 participants had a mean age of 68.0 ± 8.8 years (range 52–88 years); 39 (74%) were male. The median National Institutes of Health Stroke Scale score at presentation with stroke was 2 (range 0–5). At the time of CVR scanning, 75% had hypertension, 66% had hyperlipidemia, 13% had diabetes, 30% were current smokers or had stopped within the past 12 months, and 19% consumed alcohol regularly in excess of recommended limits (table 1). Participants were scanned at a median of 92 days poststroke (range 32–1768 days). We did not see differences in CVR values between those scanned at less than 100 days compared with those scanned at more than 100 days since stroke. Thirty-six participants (67.9%) had a final stroke subtype of lacunar, and 14 (26.4%) had discordant clinical and imaging stroke subtypes.

**Table 1:**
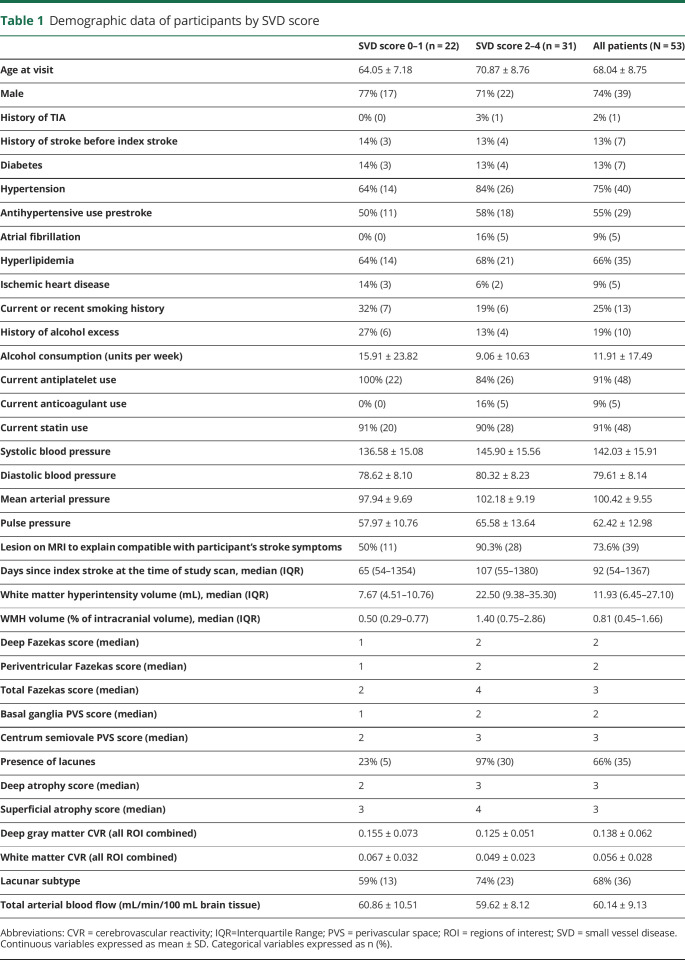
Demographic data of participants by SVD score

Imaging features of SVD were common: 27 patients (51%) had deep Fazekas score ≥2, and 29 (55%) had periventricular Fazekas score ≥2, indicating moderate to severe WMH; 17 (32%) had basal ganglia PVS score ≥3, and 28 (53%) had centrum semiovale PVS score ≥3, indicating high PVS visibility; 35 (66%) had lacunes; and 8 patients (15.1%) had microbleeds.

### Univariate analysis

#### CVR and patient characteristics

Higher systolic BP and pulse pressures were associated with lower CVR in white matter (all *p* = 0.01) and gray matter (all *p* = 0.01; data available from Dryad [table e-2, doi.org/10.5061/dryad.xpnvx0kb3]). Lower white matter CVR with increasing age did not reach statistical significance (*p* = 0.09). We found no other statistically significant associations with lower CVR.

#### CVR and SVD features

**Figure 1:**
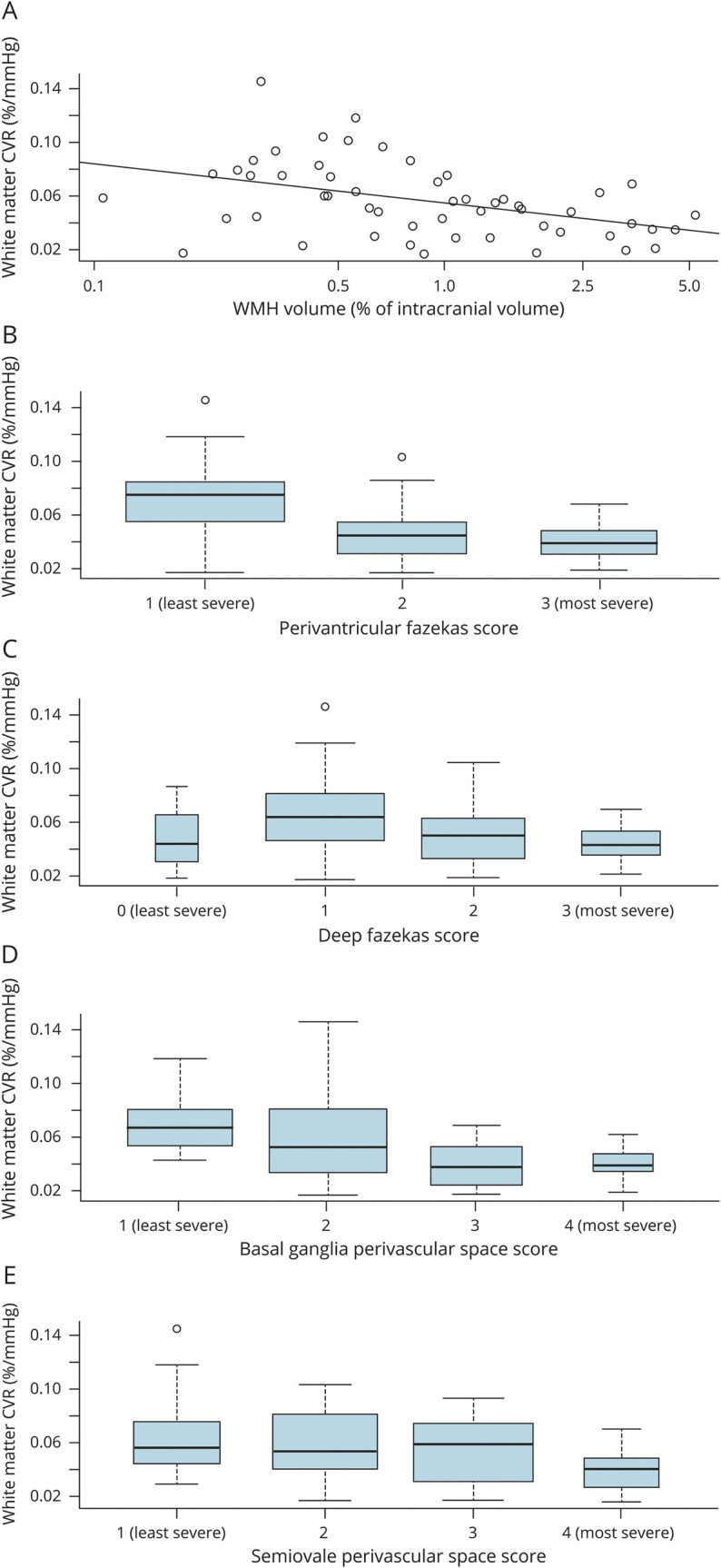
White matter CVR and WMH volume, WMH Fazekas score, and PVS scores (A) White matter CVR and WMH volume β = −0.013 (95% CI −0.020 to −0.005). (B) White matter CVR and periventricular Fazekas score β = −0.015 (95% CI −0.024 to −0.007). (C) White matter CVR and deep Fazekas score β = −0.008 (95% CI −0.017 to 0.001). (D) White matter CVR and basal ganglia PVS score β = −0.012 (95% CI −0.020 to −0.005). (E) White matter CVR and centrum semiovale PVS score β = −0.009 (95% CI −0.016 to −0.001). CI = confidence interval; CVR = cerebrovascular reactivity; PVS = perivascular space; WMH = white matter hyperintensity.

Numerous SVD features were associated with lower white matter CVR (figure 1; data available from Dryad [table e-2, doi.org/10.5061/dryad.xpnvx0kb3]): higher WMH volume, worse WMH Fazekas scores, more PVSs, presence of lacunes, deep atrophy, and total SVD score (all *p* < 0.05). Lower gray matter CVR was only associated with periventricular WMH Fazekas score (*p* = 0.03) and basal ganglia PVS (*p* = 0.05).

#### CVR, pulsatility, and CBF

**Figure 2:**
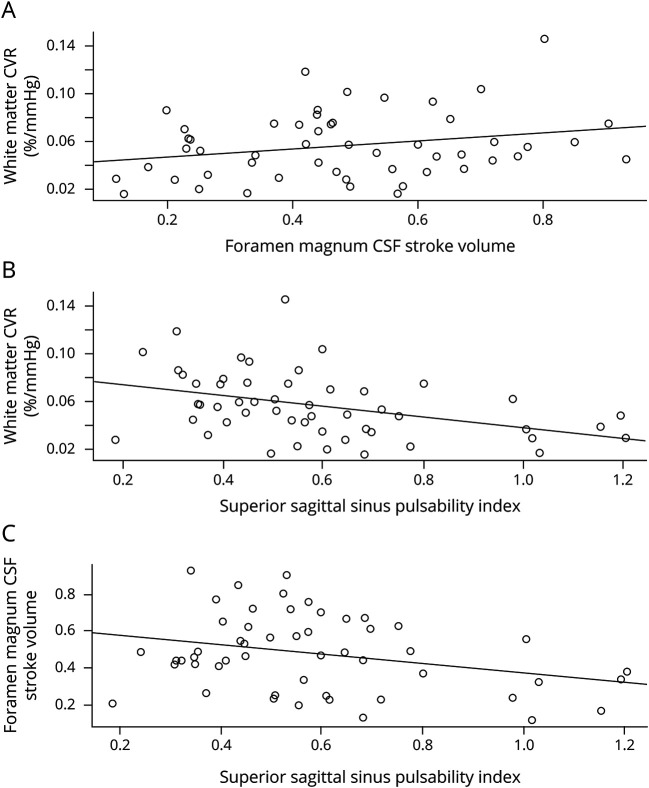
Relationship of white matter CVR, foramen magnum CSF stroke volume, and superior sagittal sinus pulsatility index (A) White matter CVR and foramen magnum CSF stroke volume β = 0.033 (95% CI −0.004 to 0.070). (B) White matter CVR and superior sagittal sinus PI β = −0.044 (95% CI −0.073 to −0.014). (C) Foramen magnum CSF stroke volume and superior sagittal sinus PI β = −0.249 (95% CI −0.477 to −0.020). CI = confidence interval; CVR = cerebrovascular reactivity; PI = pulsatility index.

Lower white matter CVR was associated with higher PI and RI in the superior sagittal, straight, and TSs (all *p* < 0.05), but not with ICA or IJV PI or RI, or with CBF, (figure 2; data available from Dryad [table e-2, doi.org/10.5061/dryad.xpnvx0kb3]). We found no univariate associations between CSF pulsatility or flow measures and CVR.

### Covariate-adjusted analyses

#### CVR and SVD features

After controlling for age, systolic BP, and sex, we found that lower white matter CVR remained associated with WMH volume (−0.01%/mm Hg change in EtCO_2_ per log10 increase in WMH volume, *p* = 0.02), periventricular WMH Fazekas score (−0.01%/mm Hg change in EtCO_2_ per point increase, *p* = 0.02), and basal ganglia PVS score (−0.01%/mm Hg change in EtCO_2_ per point increase, *p* = 0.02). The associations between these SVD features and gray matter CVR were no longer significant (table 2). We observed a similar pattern when pulse pressure was substituted for systolic BP (data not shown).

**Table 2:**
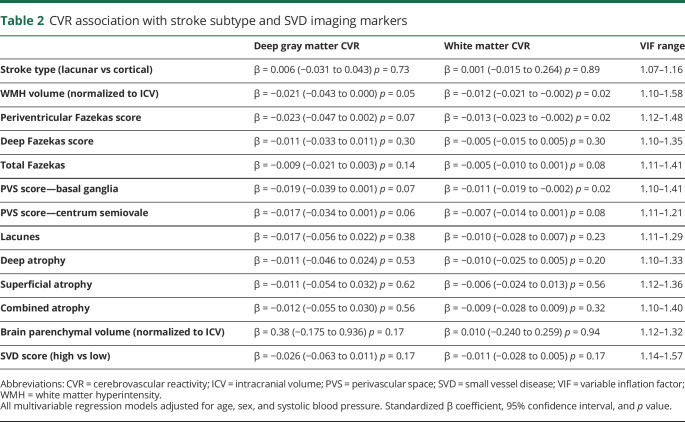
CVR association with stroke subtype and SVD imaging markers

Deep and total Fazekas scores, centrum semiovale PVS, lacunes, any brain atrophy, and total SVD score were not associated with lower CVR in either white or gray matter (table 2).

We tested the independence of the basal ganglia PVS score association with lower white matter CVR further by adding WMH volume to the regression model, which resulted in the association no longer being significant (data available from Dryad [table e-3, doi.org/10.5061/dryad.xpnvx0kb3]).

#### CVR, pulsatility, and CBF

After correcting for age, SBP, and sex, lower white matter CVR remained associated with higher SSS PI (−0.03%/mm Hg change in EtCO_2_ per unit increase in the PI *p* = 0.02), SSS RI (−0.07%/mm Hg change in EtCO_2_ per unit increase in the RI, *p* = 0.03), and IJV PI (−0.01%/mm Hg change in EtCO_2_ per unit increase in the PI, *p* = 0.04), table 3.

**Table 3:**
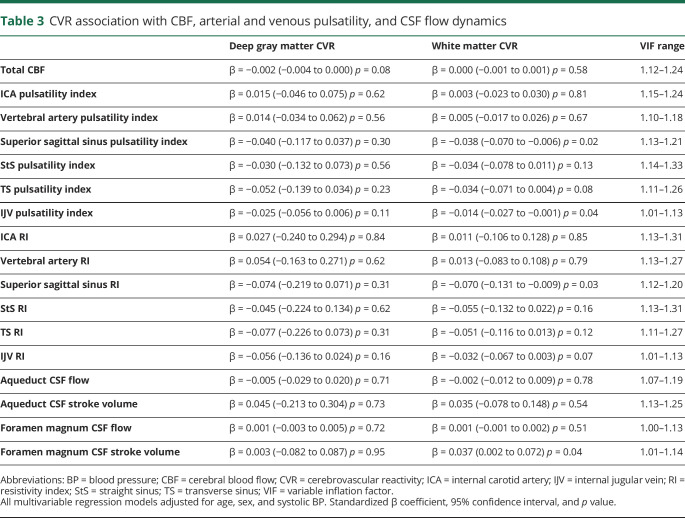
CVR association with CBF, arterial and venous pulsatility, and CSF flow dynamics

Lower white matter CVR was also associated with lower CSF stroke volume at the foramen magnum (−0.04%/mm Hg change in EtCO_2_ per mL decrease in CSF stroke volume, *p* = 0.04). Furthermore, lower CSF stroke volume was associated with higher basal ganglia PVS score (−0.96 mL per point increase in the PVS score, 95% confidence interval −2.087 to 0.146, *p* = 0.09, data available from Dryad [table e-4, doi.org/10.5061/dryad.xpnvx0kb3]). We observed a similar pattern when pulse pressure was substituted for systolic BP (data not shown). There was no association of white or gray matter CVR with arterial PI or RI or with CBF (table 3).

We further tested the association of SSS and IJV pulsatility metrics by adding WMH volume into the regression model. IJV (but not SSS) PI remained associated with lower white matter CVR (−0.01%/mm Hg change in EtCO_2_ per unit increase in the PI, *p* < 0.05, data available from Dryad [table e-3, doi.org/10.5061/dryad.xpnvx0kb3]).

## Discussion

We measured concurrently several indices of intracranial microvascular function in patients with features of SVD on neuroimaging who presented with minor ischemic stroke. We demonstrate that lower CVR is associated with higher WMH burden and higher basal ganglia PVS, independently of age, sex, and BP. The association was stronger for white matter than for subcortical gray matter CVR. Furthermore, the association between lower CVR and WMH was stronger for periventricular than deep WMH, perhaps reflecting more periventricular vulnerability to hemodynamic changes at distal end arteries, or differing pathogeneses of WMH by brain region, providing in vivo support in humans for 2 long-standing hypotheses. We also demonstrated that lower white matter CVR was associated with higher intracranial vascular pulsatility, most clearly seen in the intracranial venous sinuses and IJV, and with increased PVS visibility.^22^ Importantly, we also found that lower CVR was associated with lower CSF stroke volume at the foramen magnum, and also that lower CSF stroke volume was associated with a trend toward more basal ganglia PVS, showing for the first time in the intact human cranium, a possible link between dysfunctional brain interstitial fluid drainage (evidenced by increased PVS visibility) and impaired CSF pulsation (thought to help flush interstitial fluid through the glymphatic system).^32^ The changes we saw in CVR were small in absolute terms; however, the relative differences in CVR were often in the range of 20%–50%. The clinical relevance of this magnitude of CVR impairment requires further studies using clinical outcomes. We observed no association of lower CVR in white or gray matter with resting CBF or of resting CBF with any measures of SVD.

We recruited patients representative of the range of SVD features present in patients with typical lacunar or minor cortical ischemic stroke to ensure relevance to patients who are commonly affected by SVD.^33,34^ Confirming that vascular function changes in SVD are not confined to 1 stroke type increases the generalizability of our findings. Stroke type and SVD imaging features were carefully assessed by experienced specialists using standardized, validated image processing and analysis techniques. CVR was measured by a robust and standardized technique with quantified reliability and reproducibility.^8^ We were careful to control the statistical analyses for key patient characteristics and WMH volume where appropriate, all guided by a professional statistician.

There are limitations. Although this study is one of the largest in the literature to assess CVR in SVD^4^ and the only study so far in humans to assess CVR, vascular and CSF pulsatility, and CBF simultaneously, the sample size limits the number of comparisons and adjustment variables, and any result should be considered for clinical plausibility. We reported all results for transparency and to aid interpretation. We recruited more male than female participants, similar to previous mild stroke cohorts in our region. There is also an inherent selection bias in studies using complex imaging techniques and longer scan lengths, as only participants at the milder end of the disease spectrum and with less severe comorbidities are agreeable to participation and can tolerate the procedures. We used a 1.5T MRI scanner; however, the main impact of this vs a 3T scanner is less crisp structural resolution of the BOLD MRI image, which may affect the precision of tissue localization, but not signal magnitude.^35^ However, because we registered all the images into a common image processing space and used ROIs obtained from high-quality structural images mapped onto the CVR image to extract the tissue-specific CVR signals, the 1.5T MRI is unlikely to have affected the tissue associations.

We found a strong association of lower CVR with WMH visual score and volume. However, the Fazekas score allowed us to detect the novel finding of a stronger association between lower CVR and periventricular than deep WMH. This could reflect reduced vascularity of the distal perforating arterioles making periventricular tissue more vulnerable to developing SVD. This is consistent with recent findings in 2 similar populations where direct visualization of vessel morphology in the retina, a vascular bed homologous to the cerebral microvasculature, found that reduced retinal arteriolar branching complexity was associated with WMH and other SVD features.^36^ The finding of a stronger association between lower CVR and periventricular rather than deep WMH should be replicated in future studies, which should examine regional WMH rather than just total volume, because we show that total volume may obscure important differences in relationships between SVD lesions and microvascular function.

Associations between CVR and WMH in previous studies have varied. Lower CVR has been found in association with higher WMH volume in studies of patients with Alzheimer disease^37^ and older people with WMH^38^ (total N = 58). Lower CVR has been associated with higher visual rating measures of WMH in patients with hereditary cerebral amyloid angiopathy with severe WMH and microbleeds,^39^ patients with stroke, and patients older than 50 years with WMH with neurologic symptoms^9–12^ (total N = 152). Areas of normal white matter with lower CVR have also been shown to precede development of WMH.^11^

Of the 3 studies (total N = 197) that have shown no association between CVR and WMH, it is notable that the WMH burden of the participants was low.^40–42^ One of these studies also used a CVR method that is very different to all other studies published in the literature.^42^ In terms of age range of included participants, WMH burden, presence of clinically evident vascular disease, and CVR methodology, our study is more similar to the studies that demonstrated associations of lower CVR with more WMH.

We did not find associations of CVR with lacunes or microbleeds. This may be due to lack of statistical power but could also reflect different mechanisms driving development of different SVD imaging features.

Our systematic review highlighted the variability of CVR associations with regard to patient characteristics.^4^ The association of lower CVR with higher BP was seen in some previous studies,^40,43^ but BP associations with CVR were unclear in many other studies. A trend for increasing age to be associated with lower CVR narrowly failed to reach significance in our study, but age was associated with lower CVR in several other studies.^40,41,44,45^ The lack of a definite age-CVR relationship in the present study may reflect poorer brain vascular health in the younger compared with the older patients with stroke because patients who experience a stroke aged in their 40s or 50s may have worse vascular health than those who do not experience stroke until their ninth decade. This paradox has almost certainly dampened the expected age effect. The studies showing age effects either studied healthy elderly^41,44^ or vascular disease participants over a younger, narrower,^40^ age range than this study, missing out the effects of the arguably healthier older participants.

Lower CVR may be associated with other markers of vascular health and symptoms. Lower CVR was associated with raised inflammatory markers^46^ and more rapid decline in gait speed in patients with diabetes^47^ and with decreased insulin sensitivity in obese individuals with insulin resistance.^48^ These suggest that inflammation may drive CVR impairment and that more sensitive clinical markers of reduced CVR and SVD should be evaluated. The literature on CVR and cognitive performance is inconclusive.^49^

Higher middle cerebral artery pulsatility has previously been associated with WMH on TCD.^6^ We have shown associations of venous sinus pulsatility with WMH and PVS^22^ and now extend that finding to show association of more pulsatile blood flow with lower CVR. Higher pulsatility reflects a stiffer vascular bed. Histology in SVD reveals arteriolar sclerosis. Much less is known about venous changes, but collagenosis of the venular wall is described. In combination, lower CVR and higher pulsatility reflect a less adaptive and compliant vascular bed. This potentially results in higher shear forces being generated by blood flow and often results in a peak blood flow waveform that while higher is also shorter and therefore potentially reduces blood flow over the whole cardiac cycle. The association of increased PVS with lower CVR, in addition to higher intracranial vascular pulsatility, has not been described previously.^22^ Visible PVSs are associated with hypertension, increasing age, systemic and intracerebral inflammation, other SVD features like WMHs and microbleeds, and increased risk of dementia.^1^ Longitudinal studies are required to demonstrate whether PVS visibility precedes or follows impairments in CVR or changes in vascular or CSF pulsatility. PVSs are part of the brain's waste clearance and immune defense system, recently referred to as the glymphatic system^50^; in this regard, it is interesting that we detected altered pulsation of the CSF at the foramen magnum in association with lower CVR and with worse PVS because in experimental models, reduced PVS flushing may lead to failed clearance of metabolic debris and PVS dilation.^32,50^ Lower CSF stroke volume could indicate reduced CSF flux around the base of the brain with reduced CSF movement linked to impaired PVS flushing. Caution is required, however, because the PVS-CSF stroke volume and CVR associations could be a coassociation with other SVD features, a common problem in SVD research^30^ and should be evaluated further.

We did not find an association between WMH and CBF, consistent with previous data.^3^ However, we provide more support for a vascular dysfunction in SVD: CVR is a measure of dynamic vessel function, with impairment reflecting an inability to increase blood flow when required. The mechanisms behind this may encompass impaired endothelial function, reduced nitric oxide bioavailability, altered vascular smooth muscle function, inflammatory exudates damaging the vessel wall, and PVS function.^51^ More longitudinal data with contemporaneous vascular function measures and careful adjustment for covariates are required to unpick this pathway, but it is notable that all the associations of demographic and SVD features with impaired CVR were stronger in the white matter than the gray matter despite white matter CVR being around one-third the magnitude of that in gray matter and white matter CBF being around half of that in gray matter.^3,22^ The directionality of CVR changes in gray matter is similar to white matter, however, and therefore, lack of statistical significance may reflect lack of power in this sample. Studies of SVD mechanisms and potential interventions should consider using measures of dynamic vascular function such as CVR or pulsatility rather than resting CBF.^39,52^

We show novel and independent associations between SVD features and several measures of impaired cerebral hemodynamics, providing new insights into mechanisms that may underlie SVD development. Further longitudinal studies, controlling for confounders (e.g., age, BP, and WMH volume) will define these changes and delineate the pathway of SVD development, including the novel observation of impaired CSF pulsation. Ultimately, enhanced knowledge of vascular malfunction will help identify therapeutic targets to halt or even reverse disease progression, with benefits for both dementia and stroke prevention.

## Glossary

BP: blood pressure
BOLD: blood oxygen level dependent
CBF: cerebral blood flow
CVR: cerebrovascular reactivity
EtCO_2_: end-tidal CO_2_
FLAIR: fluid-attenuated inversion recovery
IJV: internal jugular vein
ICA: internal carotid artery
PVS: perivascular space
PI: pulsatility index
RI: resistivity index
ROI: region of interest
SSS: superior sagittal sinus
SVD: small vessel disease
StS: straight sinus
TCD: transcranial Doppler
TS: transverse sinus
VA: vertebral artery
WMH: white matter hyperintensity
